# Single-cell sequencing reveals dynamic immune features of paraneoplastic pemphigus in a patient with follicular lymphoma

**DOI:** 10.3389/fimmu.2026.1733718

**Published:** 2026-03-18

**Authors:** Rong Wei, Bochao Liu, Yun Liu, Wenjing Li, Zhiguo Chen, Jin Lu, Yuxuan Zheng, Shenmiao Yang

**Affiliations:** 1 Peking University People’s Hospital, Peking University Institute of Hematology, National Clinical Research Center for Treatment of Hematologic Disease, Beijing, China; 2 Cell Therapy Center, Beijing Municipal Geriatric Medical Research Center, Beijing, China; 3 Xuanwu Hospital, Capital Medical University, National Clinical Research Center for Geriatric Diseases, and Key Laboratory of Neurodegenerative Diseases, Ministry of Education, Beijing, China; 4 Department of Hematology, Weifang People’s Hospital, Shandong Second Medical University, Weifang, Shandong, China; 5 Center of Neural Injury and Repair, Beijing Institute for Brain Disorders, Beijing, China; 6 Center of Parkinson’s Disease, Beijing Institute for Brain Disorders, Beijing, China; 7 Human Phenome Institute, Pudong Hospital, Fudan University, Shanghai, China

**Keywords:** follicular lymphoma, paraneoplastic pemphigus, post-treatment immune remodeling, TCR - T cell receptor, tumor microenvironment

## Abstract

**Background:**

Paraneoplastic pemphigus (PNP) is a highly fatal autoimmune blistering disease that commonly occurs in patients with underlying benign or malignant neoplasms. It poses significant challenges for diagnosis and treatment. To date, the cellular and molecular mechanisms underlying the pathogenesis of PNP remain largely unclear.

**Objective:**

This study aims to elucidate the cellular and molecular mechanisms of PNP, particularly when it occurs secondary to lymphoma, by analyzing the dynamic immune landscape throughout the course of treatment.

**Method:**

We performed single-cell transcriptome sequencing and single-cell T cell receptor (TCR) analysis on peripheral blood mononuclear cells (PBMCs) and bone marrow cells (BMCs) obtained from a patient with follicular lymphoma (FL) accompanied by PNP. Samples were collected at three critical time points: before treatment, during treatment, and after successful treatment.

**Result:**

Using public datasets as a qualitative reference, we exploratorily compared patient cell frequencies to published healthy controls. In these exploratory contrasts, we observed an apparent relative abundance of *ITGAL*^+^ T cells, *TRDV1*-biased γδT-cell clusters, and *BCL2*^+^ B cells in PBMCs. In BMCs, we noted apparent differences including naïve T cell and certain B cell clusters. The single-cell transcriptome results described the lymphoma-associated tumor microenvironment and revealed post-treatment immune remodeling. This reconstitution involved activated DNA damage and T cell immune responses, which was further supported by the observation of a gradually expanded TCR clone as the treatment progressed.

**Conclusion:**

Our study delineates the dynamic immune landscape in a patient with FL-associated PNP throughout treatment. This is a descriptive, hypothesis-generating single-patient study; findings are exploratory, not generalizable, and do not establish causality. The observed changes are compatible with post-treatment immune remodeling involving specific T cell responses and TCR clone expansion. This longitudinal single-cell analysis describes the immune landscape observed in a patient with FL-associated PNP and reveals dynamic features associated with treatment response, providing a resource and generating hypotheses for future study.

## Introduction

Paraneoplastic pemphigus (PNP), first described in 1990, shares clinical and immunologic similarities with classic pemphigus vulgaris and pemphigus foliaceus (1). Subsequently, the concept of paraneoplastic autoimmune multiorgan syndrome (PAMS) emerged, highlighting the heterogeneous signs and symptoms of PNP, including severe desquamative stomatitis, polymorphous cutaneous eruptions, and multi-organopathy with progressive respiratory failure (2–4). In fact, PNP is a highly fatal autoimmune-mediated mucocutaneous disease associated with underlying neoplasm, most commonly non-Hodgkin lymphoma or chronic lymphocytic leukemia (5, 6). Mortality rates of PNP can be as high as 90% due to malignancy, sepsis, or even a frequent irreversible complication bronchiolitis obliterans (2).

PNP is an exceptionally rare disease with a low incidence rate. This rarity results in limited clinical experience and research data, which often leads to delayed diagnosis and treatment strategies based primarily on case reports and small-scale studies (7, 8). However, the challenging treatment of PNP is not only due to its rarity, but also because of the multisystem involvement, and the lack of effective treatment options (9, 10). In addition to severe skin and mucosal lesions, PNP can also affect the lungs, gastrointestinal tract, and other organs, with obliterative bronchiolitis (BO) being its most severe complication, carrying a poor prognosis and currently lacking effective treatment (11–13). Current management relies heavily on tumor control (e.g., surgical resection, chemotherapy) and immunosuppressive therapies (e.g., corticosteroids, rituximab) (12–16). However, these approaches have limited efficacy and significant side effects, including increased infection risks, drug toxicity, and the irreversible nature of pulmonary complications. Intravenous immunoglobulin (IVIG) might be helpful, but the efficacy is not confirmed. Although emerging therapies like targeted treatments and gene therapy are under investigation, they have not yet been widely applied in clinical practice. Therefore, the treatment of PNP not only requires multidisciplinary team collaboration but also focuses on psychological support and long-term management to improve prognosis and quality of life.

Though the mechanism associated studies indicated that both humoral and cell-mediated immunity contribute to the pathogenesis of PNP, the main findings showed that affected patients develop variable autoantibodies to antigens, including plakin family proteins on keratinocytes and the basement membrane zone (17–19). However, the underlying T cell population-related mechanisms have yet to be clarified.

Preliminary single-cell findings from this case were previously reported in abstract form at ASH 2025 (20). This full manuscript expands on those preliminary observations with comprehensive multi-timepoint PBMC profiling, TCR repertoire analysis, and detailed methodological reporting.

Herein, in the process of the successful diagnosis and therapeutics of a patient with follicular lymphoma (FL) accompanied by PNP, we examined the single-cell transcriptional landscape and single-cell T cell receptor data of longitudinally collected peripheral blood mononuclear cells (PBMCs), in addition to bone marrow cells (BMCs) in the patient. The longitudinal, high-resolution profiling in this rare case provides a detailed description of immune dynamics in FL-associated PNP, offering a valuable descriptive dataset and generating hypotheses for understanding disease immunopathology. It should be noted, however, that this is a descriptive, hypothesis-generating single-patient study; its findings are exploratory, not generalizable, and do not establish causality.

## Methods

### Clinical information and sample collection

Blood samples were collected from patient at various time-points after hospitalization. Sample collection, processing, and laboratory testing were conducted complied with the standard guidance. All clinical information including demographic data, medical history, symptoms, signs, and laboratory data were collected from patient medical records. The laboratory data includes blood routine, lymphocyte subsets, infection-related biomarkers, inflammatory cytokines. The total number of leukocytes, percentage of neutrophils and percentage of lymphocytes in peripheral blood were counted using hemocytometer. The number and percentage of lymphocyte subsets were analyzed using the FACSCanto flow cytometer. C-reactive protein and lactate dehydrogenase were detected by the Beckman automatic biochemical analyzer. Interleukin 6 (IL6) and other cytokines were detected using the ROCHE Elecsys assay. The study was approved by the Ethics Committees of the Peking University People’s Hospital.

### Collection of peripheral blood mononuclear cells and bone marrow cells

PBMCs and BMCs were isolated from EDTA anticoagulated venous blood and bone marrow of the patient, respectively, using a FicollR Paque Plus (Sigma Aldrich) solution according to standard density gradient centrifugation methods. Cells were harvested and counted via Cellaca MX high-throughput cell counter (Nexcelom Bioscience). The PBMCs were resuspended in 90% fetal bovine serum (FBS, HyClone), 10% DMSO freezing media and frozen using a Nalgene Mr. Frosty Cryo 1oC Freezing Container (Thermo Fisher Scientific) in a -80°C freezer for 24 h before being transferred to liquid nitrogen for long-term storage.

### Processing single-cell multi-omics sequencing data

The raw scRNA-seq and paired scTCR-seq data were simultaneously processed using 10 × Genomics software *CellRanger*. The raw sequencing data were mapped into the human reference genome and VDJ database (GRCh38).

After obtaining processed scRNA-seq data, we considered non-empty cells with the number of detected genes expressing in at least 3 cells greater than 200 using R package *Seurat* (version: 5.0.2). We then filtered the single cells to retain those with a detected gene count between 200 and 8, 000, UMI counts between 1, 000 and 60, 000, and a mitochondrial transcript percentage below 5%. To remove doublet cells, the filtered cells underwent additional processing utilizing Python package *scrublet* (21). Specifically, for each library, doublets were individually detected based on an anticipated doublet rate of 6%. Cells that exhibited a *doubletScore* exceeding the 90% quantile threshold were subsequently eliminated. Following this refinement, only high-quality single cells, characterized by a gene detection count greater than 500 and a UMI count below 40, 000 (for PBMCs) or 50, 000 (for BMCs), were retained for further downstream analysis.

Other methods include identifying cell types and cell-type-specific major genes of PBMCs and BMCs, performing the cell enrichment analysis *R*_o/e,_ identifying DEGs between pre- and post-treatment, calculating the gene module score of hallmarks, processing and analysis of scTCR-seq data. The details are specified in the **Supplementary Materials**.

## Results

### Clinical manipulation of the case

**Figure 1:**
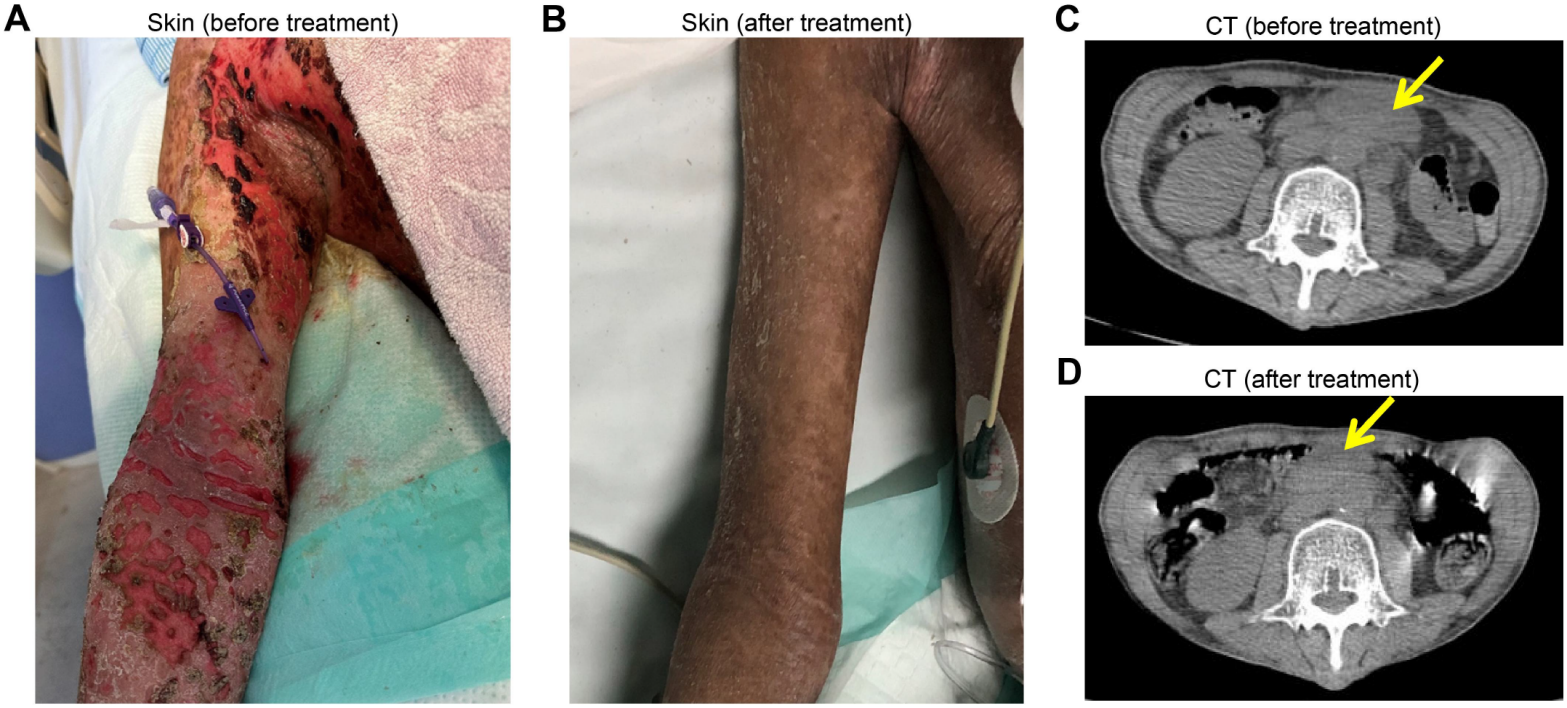
Skin condition and CT scan pre- and post-treatment. **(A)** Massive cutaneous eruption was noticed in upper extremities before treatment. **(B)** Skin lesion was improved after treatment. **(C)** CT scan showed abdominal mass before treatment. **(D)** CT scan showed reduction of the abdominal mass after treatment.

A 41-year-old male patient presented to the outpatient clinic with generalized exfoliative skin lesion. Two months prior, the patient noticed non-healing ulcers in oral and external genital mucosa. Later on, he felt discomfort in the eyes, and found blisters on the extremities. Soon he suffered from massive painful cutaneous eruption. The physical examination was notable for the skin lesion (**Figure 1A**) and enlarged lymph nodes in the neck, axillae and groin bilaterally. Laboratory tests showed normal CBC (WBC 7.65×10^9^/L, Hb 157g/L, PLT 314×10^9^/L), LDH (144U/L), and elevated levels of Ferritin (577.1ng/mL), ESR (17mm/1h), CRP (59mg/L), and IL6 (23.4pg/mL). The anti-nuclear antibody, anti-desmoglein 3 antibody, anti-bullous pemphigoid 180 antibody and anti-acetylcholine receptor antibody were positive. The biopsy of inguinal lymph node showed abnormal B cells with follicular pattern, expressing CD20, CD10, Bcl2, Bcl6 (dim), CD21 (FDC+), and Ki67 (10-20%), and negative for CD3, CD5, and cyclinD1, with EBER negative. The same abnormal immunophenotypic B cells were also detected in the bone marrow. Positron Emission Tomography/Computed Tomography (PET-CT) showed multiple lymph node involvements above and below the diaphragm (data not shown). In addition, the lips and oral mucosa, as well as the skin in the bilateral axillary regions and the proximal upper limbs, exhibited localized thickening with mildly increased glucose metabolism. The patient was diagnosed of FL with PNP and was then administered with Obinutuzumab and Bendamustine (GB regimen). After three cycles of immunochemotherapy, PET-CT evaluation achieved partially metabolism remission (data not shown). The laboratory tests found that the autoantibodies turn negative and skin lesions have significantly improved (**Figure 1B**). The patient continued with GB regimen for another cycle and returned hometown for the later on treatment.

**Table 1** presents the peripheral blood laboratory test data of the patient at five time points (C0, representing pre-treatment, and C1 to C4, corresponding to post-chemotherapy for each treatment cycle), reflecting the dynamic changes in the immune system, inflammatory responses, and hematological indicators during the treatment of FL. White blood cell count (WBC) and lymphocyte count (L) dramatically decreased after treatment at C1 and C2, suggesting potential treatment-induced immune suppression or bone marrow suppression, followed by gradual recovery at C3 and C4, indicating the possibility of post-treatment immune remodeling. Lactate dehydrogenase (LDH) levels significantly increased after treatment at C1 and C2, indicating cell destruction or tissue damage, and subsequently declined. C-reactive protein (CRP) levels decreased from 66.3 mg/L pre-treatment to 4.3 mg/L post-C4 treatment, indicating a reduction in systemic inflammation. Consistent with this trend, the cytokine IL6, which was greatly elevated pre-treatment, gradually decreased post-treatment. Other cytokines (IL2, IL4, IL10, IFNγ, etc.) were either undetectable or at low levels at most time points.

**Table 1:** Peripheral blood laboratory test data of the patient at different time points.

Time	WBC ×10^9^/L	L ×10^9^/L	Hb g/L	Plt ×10^9^/L	LDH U/L	CRP mg/L	IL2 pg/mL	IL4 pg/mL	IL6 pg/mL	IL10 pg/mL	IFNγ pg/mL	TNF αpg/mL
C0	8.18	1.59	159	320	144	66.3	<1.50	<1.50	54, 73	<2.00	<1.00	<1.00
C1	4.55	0.72	88	374	370	58.1	1.67	1.79	<1.50	2.36	<1.00	2.61
C2	2.83	1.41	71	273	345	23.6	2.83	1.80	29.54	2.51	<1.00	1.24
C3	4.24	1.64	101	307	207	27.5	2.83	2.55	26.61	5.28	<1.00	1.93
C4	6.82	1.37	115	242	265	4.3	1.77	1.61	12.15	2.06	<1.00	<1.00

Presenting the peripheral blood laboratory test data of the patient at five time points (C0, representing pre-treatment, and C1 to C4, corresponding to post-chemotherapy for each treatment cycle).

**Table 2** presents the immunological test data before treatment (C0) and after the fourth cycle of chemotherapy (C4), reflecting the dynamic changes in peripheral blood lymphocyte subsets and immunoglobulin levels during the treatment of FL. The total lymphocyte count increased from 685/uL before treatment to 1527/uL after treatment, which is compatible with potential immune recovery or post-treatment immune remodeling. The absolute count of total T cells increased (from 631/uL to 1358/uL), but their abundance slightly decreased (from 92.05% to 89.04%), indicating a proportional shift in lymphocyte subsets. The absolute count of CD4^+^ T cells showed a minimal increase (from 183/uL to 191/uL), but their abundance decreased (from 28.54% to 13.03%), suggesting a relative decline in this subset. In contrast, CD8^+^ T cells exhibited a substantial increase in both absolute count (from 389/uL to 1037/uL) and abundance (from 60.53% to 82.56%), indicating a dominant expansion of this subset. The CD4/CD8 ratio decreased from 0.47 before treatment to 0.16 after treatment, further reflecting the relative expansion of CD8^+^ T cells. B cells were nearly absent after treatment (0/uL, 0%), which is associated with the use of CD20 monoclonal antibody in the treatment regimen. Natural killer (NK) cells increased in absolute count (from 52/uL to 64/uL), but their abundance decreased (from 7.14% to 5.06%). Regarding immunoglobulins, IgG levels increased from 4.95 g/L before treatment to 7.44 g/L after treatment, suggesting enhanced or recovered humoral immunity, while IgA levels remained stable (0.92 g/L at C0 and 0.90 g/L at C4), and IgM levels were below the detection limit (<0.20 g/L) at both time points. These data collectively reflect the complex changes in the immune system and inflammatory responses during FL treatment.

**Table 2:** Immunological test data before treatment and after the fourth cycle of immunochemotherapy.

Time	Lymphocytes (/uL)	Total T (/uL, %)	CD4+ T (/uL, %)	CD8+ T (u/L, %)	CD4/CD8	B (/uL, %)	NK (/uL, %)	IgG (g/L)	IgA (g/L)	IgM (g/L)
C0	685.00	631 (92.05%)	183 (28.54%)	389 (60.53%)	0.47	1.00 (0.10%)	52.00 (7.14%)	4.95	0.92	<0.20
C4	1526.83	1358.09 (89.04%)	190.74 (13.03%)	1037.43 (82.56%)	0.16	0 (0.00%)	64.35 (5.06%)	7.44	0.90	<0.20

Presenting the immunological test data before treatment (C0) and after the fourth cycle of immunochemotherapy (C4).

### PBMC single-cell transcriptome analysis revealing an apparent relative abundance of *ITGAL*^+^ T cells and two *TRDV1*-biased γδT-cell clusters

**Figure 2:**
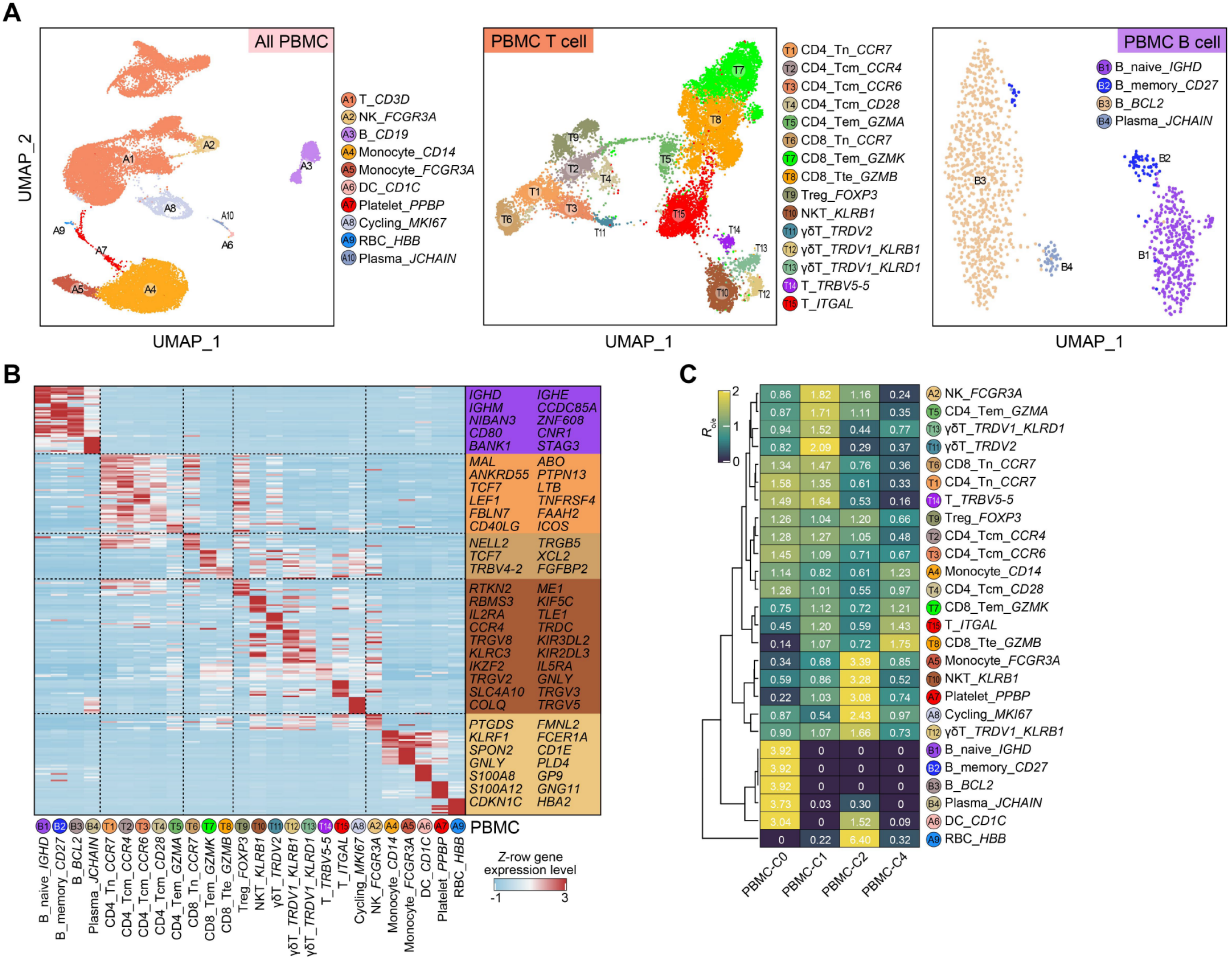
Single-cell transcriptome landscape of PBMCs from the FL associated PNP patient prior- and post-treatment. **(A)** UMAP plots showing the cell type (all immune cells, left) or transcriptionally defined clusters (T-cell clusters, middle; B-cell clusters, right) among PBMCs, including the *BCL2*^+^ *IGHG1*^+^ lymphoma-associated B-cell cluster. **(B)** Heatmap showing the row-scaled expression level of cell-type-specific marker genes among PBMCs. Representative marker genes are indicated at right. **(C)** Heatmap showing the *R*_o/e_ result among distinct cell types in PBMCs prior- and post-treatment. The *R*_o/e_ values for the “HCs” column (far left) are derived from an exploratory, qualitative comparison to an unmatched public dataset and are not suitable for formal statistical inference.

To decipher the cellular and molecular characteristics and potential mechanisms of the PNP through this case, we performed single-cell multi-omics sequencing, including single-cell RNA-seq (scRNA-seq) and single T cell receptor repertoire sequencing (scTCR-seq), using PBMCs collected from four time points prior- and post-treatment (C0, C1, C2, and C4). After cell filtering, 26, 544 single cells were retained for the downstream analysis (**Supplementary Figure 1A**). Unsupervised clustering and uniform manifold approximation and projection (UMAP) visualization analysis identified ten main immune cell types, and further re-clustering analysis identified 15 T cell subtypes and three B cell subtypes (**Figure 2A**, **Supplementary Figure S1B**). Moreover, we identified cell-type-specific marker genes, and the specific expression profiling of these marker genes also supported the cell annotation (**Figure 2B**, **Supplementary Figures S1C, D**).

We additionally collected single-cell RNA-seq data of healthy controls (HCs) from the previous study (22), and then applied the ratio of observed and expected value (*R*_o/e_) analysis which was performed within a *Chi*-square testing framework as the previous studies (23, 24). Using public datasets as a qualitative reference, we exploratorily compared patient cell frequencies to published healthy controls. Although PBMC healthy controls and the patient were profiled on the same platform and show good alignment after Harmony integration, cross-study cell frequency comparisons remain susceptible to residual batch effects and pseudoreplication. Therefore, we treat *R_o/e_*-based differences as exploratory and hypothesis-generating only. In these comparisons, we observed apparent differences, including a relatively higher frequency of CD14^+^ monocytes and several T cell subtypes in the patient, including regulator T cell (Treg), CD4 effector memory T (Tem) cell, and natural killer T (NKT) cell, while *FCGR3A*^+^ monocyte, CD4/CD8 naïve T cell, mucosal-associated invariant T (MAIT) cell, naïve B cell, and memory B cell appeared less frequent (**Supplementary Figure 1E**). We also identified a transcriptionally defined *BCL2*^+^ *IGHG1*^+^ B-cell cluster, characterized by high expression of *BCL2* and memory B-cell–associated immunoglobulin profiles (*IGHG1*) while a relatively low expression of the plasma cell marker gene (*JCHAIN*) (**Figure 2A** and **S1F**). This cluster is consistent with the known molecular features of FL and is interpreted as representing circulating or bone marrow–resident lymphoma-associated B cells, rather than a distinct or novel B-cell subtype. Several transcriptionally defined T-cell clusters, including the *ITGAL*^+^ T cell and *TRBV5-5*^+^ T cell clusters, were enriched or uniquely observed in the patient (**Supplementary Figures 1E**, **S1G**). Gene Ontology (GO) analysis revealed that cell-type-specific marker genes of *ITGAL*^+^ T cells were associated with the response to hormone/stress/decreased oxygen levels/DNA damage, compatible with an activated or stress-associated T-cell transcriptional state observed in this patient within the tumor and treatment context (**Supplementary Figure 1H**). Notably, in exploratory contrasts with public healthy control data (HCs), γδT cells from controls typically expressed *TRDV2*, whereas in this patient we observed two *TRDV1*-biased γδT-cell clusters characterized by an apparent predominance of *TRDV1* expression and NK receptors (*KLRB1* and *KLRD1*) but minimal or even no expression of *TRDV2*.

As treatment progresses over time, the abundance of CD4/CD8 naïve T cell, *TRBV5-5*^+^ T cell, CD4 central memory T (Tcm) cell, and Treg gradually decreased, while the abundance of CD8 Tem cell and CD8 terminal effector (Tte) cell increased after 4-cycle treatment (**Figure 2C**). This observation suggests an association between increased CD8 effector and terminal effector T-cell abundance and clinical improvement in this patient, generating hypotheses regarding their potential involvement during disease resolution.

### Baseline (C0) BMC single-cell transcriptome analysis revealing the enrichment of naïve T cells and *BCL2*^+^ *IGHG1*^+^ lymphoma-associated B-cell clusters

**Figure 3:**
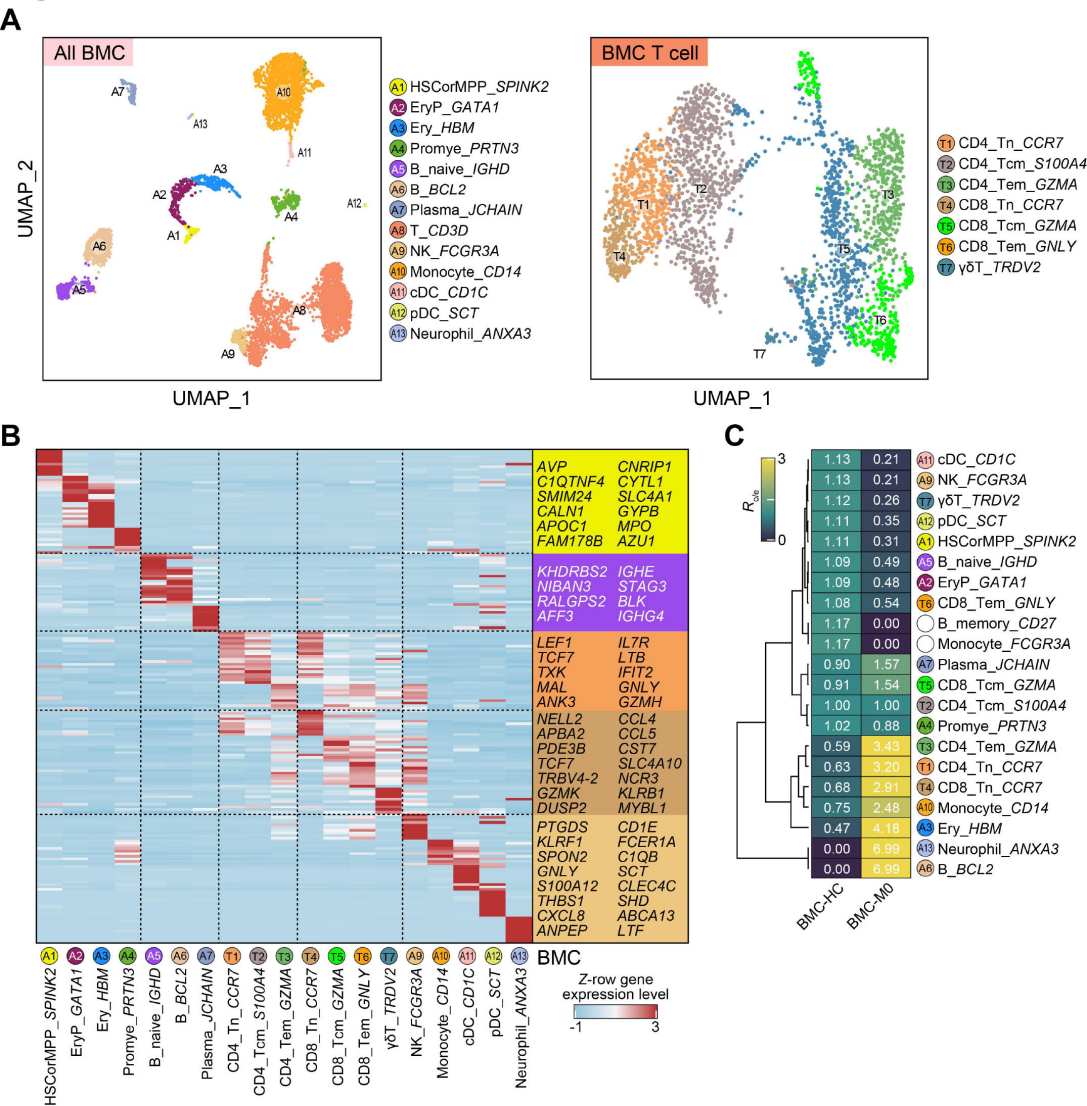
Single-cell transcriptome landscape of BMCs from the FL associated PNP patient. **(A)** UMAP plots showing the cell type (all immune cells, left) or subtype (T cell subtype, right) information among BMCs. **(B)** Heatmap showing the row-scaled expression level of cell-type-specific marker genes among BMCs. Representative marker genes are indicated at right. **(C)** Heatmap showing the *R*_o/e_ result among distinct cell types in BMCs from young HCs and the PNP patient. This is an exploratory, qualitative comparison between unmatched samples and is not intended for formal inference.

The tumor microenvironment (TME) of FL is highly complex, primarily composed of tumor cells, immune cells, stromal cells, and the extracellular matrix. Within the TME of FL, immune cells such as tumor-associated macrophages (TAMs), Tregs, and follicular helper T cells (Tfh) promote the survival and proliferation of tumor cells by secreting cytokines and chemokines. Meanwhile, these immune interactions may play a significant role in the development of complications, highlighting their critical importance in the disease’s progression and associated outcomes. Thus, we additionally collected BMCs at the baseline time point (C0) prior to treatment initiation, which were then subjected to single-cell RNA sequencing. After cell filtering, 6, 856 single cells were applied for the unsupervised clustering, and we then identified 13 major cell types and seven T cell subtypes (**Figure 3A**, **S1A**). We also identified cell-type-specific marker genes for these cell types (**Figure 3B**).

To examine the altered composition of the bone marrow microenvironment in the patient, we obtained the public single-cell RNA-seq data of BMCs from young HCs (25). Exploratory comparison to a public dataset of healthy bone marrow using *R*_o/e_ analysis suggested contrasts in cellular composition. In the patient, several innate immune cells, memory B cells, and naïve B cells appeared less frequent relative to the public healthy control data (HCs), while CD4/CD8 naïve and Tem cells appeared more frequent (**Figure 3C**), in line with the cellular composition of PBMCs described as above. These findings are compatible with the possibility that the altered cellular composition of the bone marrow microenvironment may be related to the alteration of PBMC composition.

### Activated DNA damage and immune responses together triggering post-treatment immune remodeling

**Figure 4:**
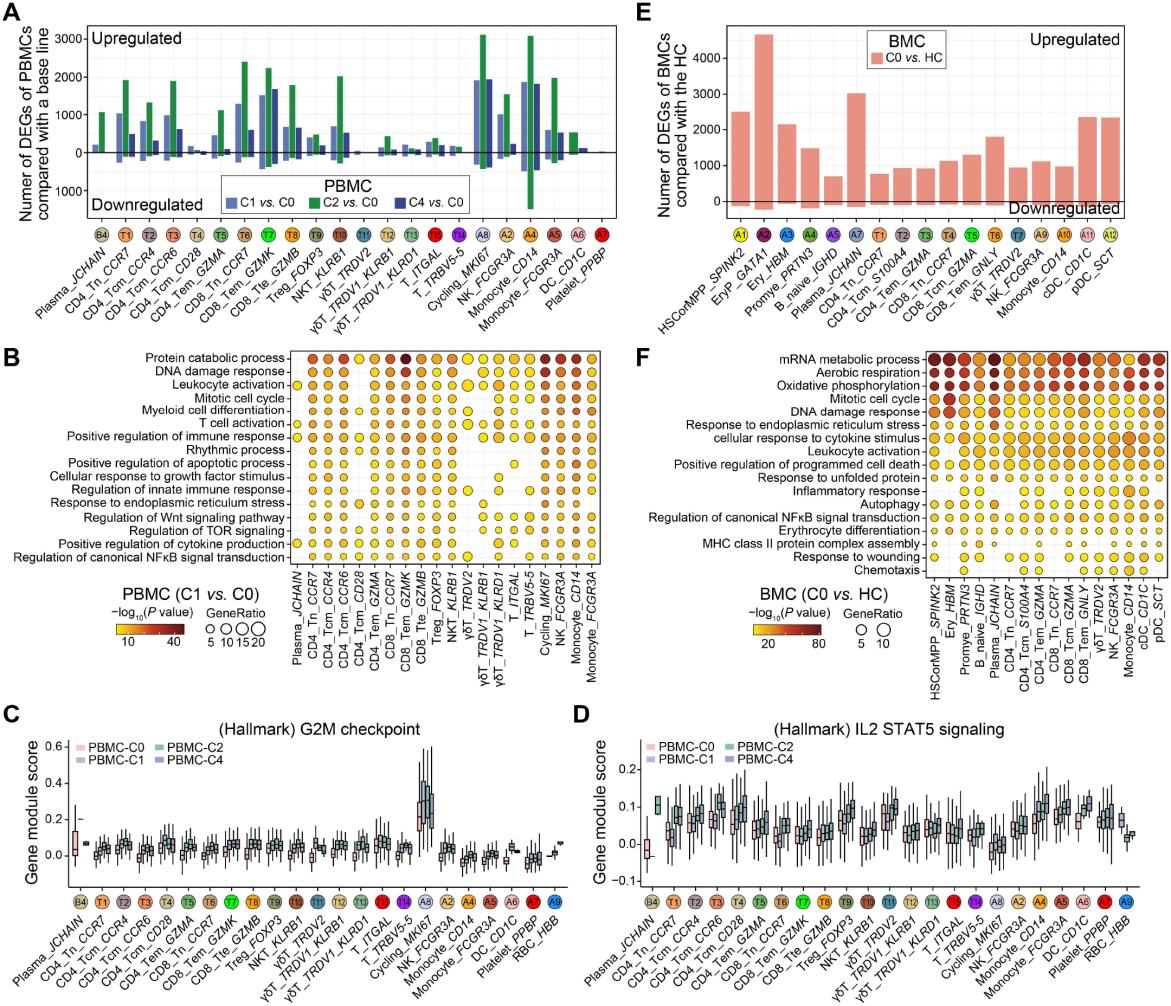
Transcriptome characteristics of PBMCs and BMCs from the FL associated PNP patient. **(A)** Barplot showing the number of DEGs between distinct treatment time points and a base line (prior-treatment) in PBMCs from the PNP patient. **(B)** Dot plot showing GO terms of upregulated DEGs in PBMCs from the FL associated PNP patient, comparing the state after 1-cycle treatment to the baseline. Dot size indicates the ratio of related genes among inputs and dot color indicates the statistical significance. **(C, D)** Boxplots showing the gene module score of hallmarks of G2M checkpoint **(C)** and DNA repair **(D)** among PBMCs prior- (C0) and post-treatment (C1, C2, C4). **(E)** Barplot showing the number of DEGs identified in an exploratory comparison between the patient’s baseline (C0) BMCs and a public dataset of bone marrow from public young healthy control data (HCs). **(F)** Dot plot showing Gene Ontology (GO) terms enriched among the DEGs from the exploratory comparison described in **(E)**. Dot size indicates the ratio of related genes among inputs and dot color indicates the statistical significance. These comparisons are qualitative and hypothesis-generating due to cross-dataset variability.”.

Given the single-patient design and the use of cell-level differential testing, the following DEG- and GO-based signatures should be viewed as descriptive trends rather than formally validated differential expression. In an exploratory DEG analysis comparing the patient’s PBMCs to public healthy control data (HCs), transcriptome differences were most notable in CD4 and CD8 T cells and monocytes (**Supplementary Figure 2A**). These comparisons are qualitative and hypothesis-generating due to inherent cross-dataset variability. Given the improvement in disease state following treatment, we subsequently compared the transcriptional alterations prior- and post-treatment to elucidate the potential molecular mechanisms to underlying disease progression and recovery. As similar as transcriptional differences compared to HCs, we identified thousands of upregulated DEGs prior- and post-treatment and these DEGs mainly enriched in T cell subtypes, NK cells, and CD14^+^ monocytes (**Figure 4A**). However, relative fewer downregulated DEGs were identified after treatment, except for CD14^+^ monocytes.

In this descriptive framework, GO analysis suggested that genes enriched in activated immune responses, such as leukocyte activation (i.e., *FCGR3A, IL7R*, and *STAT5B*), T cell activation (i.e., *JAK1, CLEC7A*, and *ANXA1*), and positive regulation of cytokine production (i.e., *CEBPB, IFNGR1*, and *CD86*), were upregulated post-treatment (**Figure 4B**, **S2B**). Moreover, mitotic cell cycle-related genes were also upregulated post-treatment, along with DNA damage response-related genes. When we examined the hallmark profiling, we detected the increased hallmark expression levels of G2M checkpoint and IL2 STAT5 signaling post-treatment compared to prior-treatment (**Figures 4C, D**). Additionally, we noticed that DNA damage response-related genes were also upregulated post-treatment, and in line with this. (**Figure 4B**, **S2C**).

We further identified DEGs between the patient and HCs in BMCs, and revealed thousands of upregulated DEGs (**Figure 4E**). Similar to PBMCs, the upregulated DEGs enriched in DNA damage response, cellular response to cytokine stimulus, leukocyte activation, position regulation of programmed cell death, and chemotaxis (**Figure 4F**).

### A gradually expanded TCR clone as treatment progresses over time

**Figure 5:**
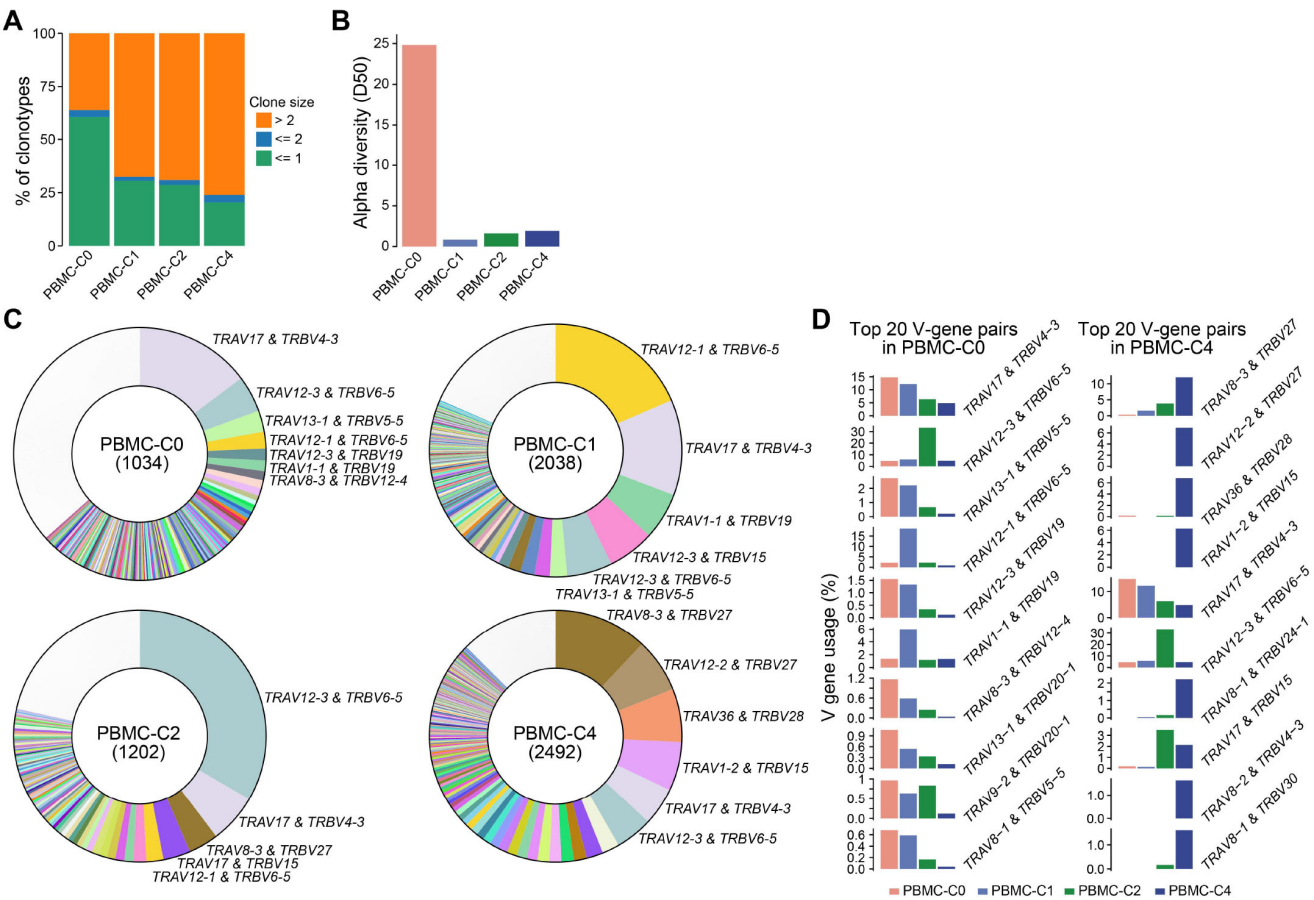
Single-cell TCR repertoire of the PNP patient. **(A)** Stack barplot showing the percentage of TCR clonotypes with distinct clone size prior- and post-treatment. **(B)** Barplot showing the TCR diversity prior- and post-treatment. **(C)** Pie charts showing the percentage of distinct V-gene pairs in α- and β-chains prior- and post-treatment. The number of detected TCRs is indicated in the bracket. The top-ranked V-gene pairs are indicated. **(D)** Barplots showing the percentage of top-ranked V-gene pairs.

Upon analyzing scTCR-seq data, we found an observed distinction in the TCR repertoire prior- and post-treatment, implying that the treatment modulated the global TCR repertoire (**Supplementary Figure 3A**). We observed a gradual accumulation of expanded clones over the course of treatment, with a notable surge after 4-cycle treatment (**Figure 5A**). Consistently, the TCR diversity was detected to be high prior to treatment, but it underwent a sharp decline following treatment (**Figure 5B**). Next, we extended our investigation to detailed clonal expansions across T cell subtypes, revealing a high frequency of expanded clones in CD4 Tem, CD8 Tem, CD8 Tte, and NKT cells, as expected (**Supplementary Figure 3B**). We noticed that the abundance of expanded clonotypes in CD8 Tte cells increased over the course of treatment. Interestingly, the cellular abundance of CD8 Tte cells also increased after treatment (**Supplementary Figure 3B**). These observations describe an increased representation of expanded clonotypes within CD8 Tte cells over the course of treatment, compatible with therapy-associated clonal focusing rather than direct evidence of disease-specific immune remodeling. Of note, the abundance of expanded clonotypes CD8 Tem cells decreased over the course of treatment, compatible with the resolving inflammation (**Supplementary Figure 3B**).

Furthermore, we examined the V-gene usage in α- and β-chains to track clonal enrichment over the treatment period. Notably, the most enriched V-gene pairs varied at different time points prior- and post-treatment (**Figure 5C**). Prior to treatment, the dominant V-gene pair was *TRAV17* & *TRBV4-3*, but its frequency decreased gradually over the course of treatment (**Figure 5D**). In contrast, *TRAV8-3* & *TRBV27* emerged as the most enriched V-gene pair post treatment, with its gene usage increasing steadily over the treatment period.

Taken together, these findings describe dynamic shifts of T cell subtypes and accumulation of expanded clones during treatment.

## Discussion

Our patient was diagnosed of FL exhibiting CD20 positive (90%). Staging of lymphoma by Ann Arbor system was IVB, involved lymph nodes above and below the diaphragm and bone marrow, with abdominal mass. The Follicular Lymphoma International Prognosis Index (FLIPI) score (26) is 2. For the treatment of patients with FL, the GALLIUM study confirmed the efficacy of the Obinutuzumab-based regimen (27). Therefore, the regimen of Obinutuzumab and Bendamustine was selected in this case. Of note, our case was associated with a rare lymphoma comorbidity, PNP, which is an autoimmune disease that involves mucous membranes and skin (28, 29). PNP has limited treatment options and a high mortality rate. In this case, the patient accepted the treatment of FL. The mid-term efficacy assessment achieved partial remission (PR), while the symptoms of PNP got controlled as well. In this patient, the improvement in PNP symptoms was observed alongside lymphoma treatment, which is compatible with the known association between the two conditions.

The pathogenesis of PNP involves both humoral and cellular immune dysregulation. While auto-antibodies targeting desmosomal proteins (e.g., desmogleins) drive epithelial blistering through direct tissue damage, dysregulated T cell subsets (e.g., CD8^+^ cytotoxic T cells and γδT cells) exacerbate inflammation and multi-organ injury via cytokine release and direct cytotoxicity (30–32). Recent studies emphasize the critical role of T cell-mediated immunity, particularly CD8^+^ cytotoxic T cells and γδT cells, in driving tissue destruction and perpetuating autoimmunity (9, 10). These T cell subsets infiltrate lesional tissues, secrete pro-inflammatory cytokines such as interferon-gamma (IFN-γ) and tumor necrosis factor-alpha (TNF-α), and induce keratinocyte apoptosis, exacerbating mucosal and cutaneous lesions (9, 32). Current therapeutic strategies prioritize controlling the underlying malignancy (e.g., lymphoma resection, chemotherapy) combined with immunosuppressive agents, such as corticosteroids, rituximab (anti-CD20), and intravenous immunoglobulins (IVIG) (10, 16). However, these treatments often fail to address T cell-driven pathology, leading to refractory disease and high mortality.

Our single-cell analysis revealed the enrichment of *ITGAL*^+^ T cells and two *TRDV1*-biased γδT-cell clusters (*TRDV1*^+^ *KLRD1*^+^ and *TRDV1*^+^ *KLRB1*^+^). *ITGAL* (also named as CD11a) is a key integrin involved in T cell adhesion and migration, facilitating immune cell infiltration into inflamed tissues (10, 33). In this PNP patient study, the enrichment of an *ITGAL*^+^ T-cell transcriptional phenotype is interpreted as reflecting an activation or stress response in the context of malignancy and chemo-immunotherapy, rather than a uniquely pathogenic or PNP-specific T-cell cluster(s). The biased γδT-cell clusters, characterized by *TRDV1* and NK receptor expression (*KLRD1/KLRB1*), diverged from healthy controls expressing *TRDV2*. γδT cells are known to bridge innate and adaptive immunity, and their dysregulation in PNP may drive aberrant cytokine production (e.g., IFN-γ) and direct epithelial cytotoxicity (34). These subsets likely exacerbate mucosal injury and perpetuate autoimmunity, aligning with reports of γδT cell involvement in PNP-associated bronchiolitis obliterans (35).

The decline in CD4^+^ naïve T cells and Tregs, coupled with the expansion of CD8^+^ Tte and Tem cells post-treatment, underscores the dominance of cytotoxic T cell responses in disease resolution. CD8^+^ Tte cells, marked by *GZMB* expression, likely mediate tumor cell clearance and suppression of autoantibody-producing B cells (36, 37). In contrast, the depletion of Tregs may reflect impaired immune regulation during active disease, permitting unchecked T and B cell activation (38). This imbalance is compatible with a proposed dual role of CD8^+^ T cells in both antitumor immunity and immunopathology in PNP.

The identification of *BCL2*^+^ B cells in both peripheral blood and bone marrow aligns with their role as follicular lymphoma cells. *BCL2* overexpression confers apoptosis resistance, enabling malignant B cell survival and shaping an immunosuppressive TME (38). These cells may directly interact with T cells, promoting Treg differentiation and CD8^+^ T cell exhaustion via cytokine secretion (e.g., IL10) (9, 38). Furthermore, *BCL2*^+^ B cells could serve as a reservoir for autoantibody production, perpetuating humoral autoimmunity in PNP (19). The near-complete depletion of B cells post-obinutuzumab treatment correlates with clinical improvement, emphasizing the centrality of B cell targeting in PNP management (16).

The longitudinal scTCR-seq data revealed a progressive shift from *TRAV17/TRBV4-3*-dominant clones pretreatment to *TRAV8-3/TRBV27*-enriched clones post-treatment. This observed clonal shift is compatible with the hypothesis that chemotherapy and anti-CD20 therapy may remodel the TCR repertoire (32). The contraction of TCR diversity and the accumulation of expanded clonotypes post-treatment are consistent with well-recognized effects of chemo-immunotherapy, including lymphodepletion followed by clonal rebound. This finding describes therapy-associated repertoire remodeling observed in this patient. However, the persistence of expanded CD8^+^ Tte clones underscores the need for sustained surveillance to prevent relapse.

This study documents a rare instance of FL-associated PNP, with thorough analysis of clinical features, immunological profiles, and tumor microenvironment. However, the single-case nature inherently restricts generalizability and statistical power. Key limitations of this descriptive study include: Firstly, the single-case, uncontrolled design precludes comparative analysis with FL cases without PNP, weakening mechanistic inferences about lymphoma-associated PNP; Potential unmeasured confounders due to minimal sample size, possibly affecting the reliability and external validity of observations. Furthermore, because DEGs were identified at the cell level without *pseudobulk* modeling, the risk of pseudoreplication is high, and effect sizes and pathway-level patterns should therefore be interpreted as hypothesis-generating only rather than confirmatory. Nevertheless, these findings establish a critical foundation for identifying disease-specific patterns and guiding future investigations. To address these limitations, subsequent research should prioritize longitudinal cohorts of FL-PNP patients with matched controls (e.g., FL patients without PNP) to validate these findings and elucidate lymphoma-specific immune mechanisms.

Secondly, frequency-based comparisons of PBMCs and BMCs to the external healthy control datasets from Stephenson et al. (2021) and Triana et al. (2021) are subject to limitations (22, 25). Despite *post hoc* bioinformatic alignment (e.g., Harmony), differences in sample processing, donor variability, and the lack of matched replication mean that residual batch effects and pseudoreplication are a concern and cannot be fully ruled out. Therefore, these comparisons should be viewed as exploratory and qualitative only, and any apparent differences require validation in future, controlled cohort studies.

In summary, our study utilized the single-cell multi-omics sequencing technology to investigate the unique immune environment in a lymphoma patient accompanied by PNP prior- and post-treatment. The *BCL2*^+^ lymphoma B cells interact with T cells; both contribute to composition of tumor microenvironment. Additionally, the dynamic changes in the transcriptome and TCR clones post-treatment are compatible with post-treatment immune remodeling. Our findings describe the tumor microenvironment of follicular lymphoma and cellular dynamics in PNP, providing a resource for future mechanistic investigations. As a descriptive, hypothesis-generating study of a single patient, our results are exploratory and not generalizable, and do not establish causality.

## Data Availability

The raw sequence data reported in this study have been deposited in the Genome Sequence Archive (GSA)-Human under accession number HRA016740. The processed scRNA-seq and scTCR-seq data reported in this study have been deposited in the Gene Expression Omnibus under accession number GSE318717. Publicly available software used in this study are listed in the method.
