# Supplementary material for: Single-cell sequencing reveals dynamic immune features of paraneoplastic pemphigus in a patient with follicular lymphoma
Source: Front Immunol. 2026 Mar 18;17:1733718. doi: 10.3389/fimmu.2026.1733718 (PMC13040559; doi:10.3389/fimmu.2026.1733718)
Supplement: Supplementary file 1 [file DataSheet1.pdf]

Supplemental material

Figure S1. Single-cell transcriptome landscape of PBMCs from the FL associated PNP patient prior- and post-treatment

(A) Violin plots showing the number of detected genes (top) and UMIs (middle), and the percentage of mitochondrial transcripts (bottom) in PBMCs (left) and BMCs (right).

(B) UMAP plots showing the time point information in immune cells (left), T cells (middle), and B cells (right) among PBMCs.

(C and D) Dotplots showing the scaled expression level of cell-type-specific marker genes among peripheral T-cell (C) and B-cell (D) clusters, including the *TRDV1*-biased γδT-cell cluster with NK receptor expression and the $BCL2^{+}$ $IGHG1^{+}$ lymphoma-associated B-cell cluster. For each gene, the gene expression level is scaled across cell subtypes.

(E) Heatmap showing the $R_{o/e}$ result among distinct cell types in PBMCs within HCs and the PNP patient.

(F) UMAP plots showing the distribution (left) and expression profiling (right) of $BCL2^{+}$ B cells among all B cells excluding plasma cells.

(G) UMAP plots showing the distribution (left) and expression profiling (right) of $ITGAL^{+}$ T-cell cluster (transcriptional phenotype enriched in the patient) among all T cells.

(H) Barplot showing GO terms of cell-type-specific marker genes of $ITGAL^{+}$ T cells.

Figure S2. Transcriptome characteristics of PBMCs and BMCs from the FL associated PNP patient

(A) Barplot showing the number of DEGs between the PNP patient and HCs in PBMCs.

(B) Dot plot showing GO terms of upregulated DEGs in PBMCs from the PNP patient, comparing the state after 2-cycle (left) and 4-cycle (right) treatment to the baseline. Dot size indicates the ratio of related genes among inputs and dot color indicates the statistical significance.

(C) Boxplot showing the gene module score of hallmarks of IL2 STAT5 signaling among PBMCs prior- and post-treatment.

Figure S3. Single-cell TCR repertoire of the FL associated PNP patient

(A) Heatmap showing the TCR similarity across time points. The similarity is measured by the Jaccard method.

(B) Stacked barplots showing the percentage of clone size class (single clonotype, and expanded clonotype with two clones or more than two clones) in each T cell subtype across time points.

Supplemental Methods

Identifying cell types and cell-type-specific major genes of PBMCs and BMCs

When we identified cell types of PBMCs and BMCs based on scRNA-seq data, we separately pre-processed each library, and applied the same pre-processing method for PBMCs and BMCs. The scRNA-seq data, quantified in terms of raw UMI counts, were imported into *Seurat* framework to distinguish between various cell types or subtypes. We standardized and scaled the UMI counts using function *NormalizeData* and *ScaleData* in *Seurat* with default parameters, respectively. We then identified high variable genes (HVGs) using function *FindVariableFeatures*, and performed principal component analysis (PCA) using function *RunPCA*. To mitigate potential batch effects, we integrated libraries according to Harmony algorithm using function *IntegrateLayers* and *JoinLayers*. We then selected the first 30 Harmony dimensions to conduct non-linear dimensionality reduction analysis via the UMAP algorithm, employing function *RunUMAP*. After that, an unsupervised clustering analysis was conducted using function *FindClusters* with a clustering parameter '*resolution*' set at 2. Finally, based on the expression patterns of well-established lineage marker genes, primary immune cell types, and T and B cell subtypes were identified and annotated.

We also identified cell-type-specific marker genes, which were

differentially expressed genes (DEGs) among the diverse cell types. In this process, the Wilcoxon rank sum test was employed utilizing function *FindAllMarkers* within *Seurat* framework. We adopted three stringent criteria for defining a gene as a DEG: firstly, a log2-transformed fold change exceeding 0.25; secondly, a false discovery rate (FDR) below 0.05; and lastly, the percentage of cells expressing that gene in the corresponding cell type surpassing 25%.

Performing the cell enrichment analysis $R_{o/e}$

$R_{o/e}$ ratios and chi-square tests were used as a descriptive heuristic to explore contrasts in cell type frequencies between samples. Because each cell is not an independent biological replicate, and library/batch effects were not formally modeled in this single-patient study, we do not treat these *p*-values as formal evidence of enrichment or depletion and do not perform cross-patient inference. For descriptive purposes within this study, we noted a $R_{o/e}$ ratio > 1 as an apparent increased frequency and a ratio < 1 as an apparent decreased frequency[1,2].

$$R_{o/e} = \frac{cell\_number_{observed}}{cell\_number_{expected}}.$$

Identifying DEGs between pre- and post-treatment

To identify DEGs between pre- and post-treatment according to scRNA-seq data, we utilized the gene expression data quantified with the

$\log_2$-transformed counts per million (CPM). The CPM was defined as the UMI count of a given gene in a given cell dividing the total UMI count of this cell and multiplying 100,000.

To identify differentially expressed genes (DEGs) between pre- and post-treatment conditions, we used the same approach as for identifying cell-type-specific marker genes: the Wilcoxon rank sum test implemented in the *FindAllMarkers* function. Specifically, the DEG analysis relied on per-cell Wilcoxon testing, performed without *pseudobulk* aggregation and without modeling sample-level covariates (time, library). This approach can inflate significance because individual cells are not independent biological replicates. Therefore, DEGs and downstream GO/enrichment analyses are interpreted descriptively as generating hypotheses, not as confirmatory statistical evidence. We defined the DEG requiring the following three conditions to be met: 1) a log2-transformed fold change exceeding 0.25, 2) an FDR less than 0.05, and 3) the percentage of cells expressing the gene in the corresponding group (pre- and post-treatment) being greater than 25%.

To gain further insights into the biological functions of these DEGs, we performed Gene Ontology (GO) analysis using web-based tool *Metascape* with default parameters.

Calculating the gene module score of hallmarks

Based on the hallmark list obtained from the GSEA website, we quantified the gene module score for each individual cell using function *AddModuleScore* within *Seurat* framework.

Processing and analysis of scTCR-seq data

To facilitate integration with our scRNA-seq data processed in *Seurat*, the scRNA-seq data of T cells was converted from '*RDS*' to '*H5DF*' format using R package *SeuratDisk* (version: 0.0.0.9021). Subsequently, only TCRs detected in the scRNA-seq data were retained for downstream analysis.

The scTCR-seq data was initially processed with *CellRanger* as described above. The resulting '*filtered_contig_annotations.csv*' file was then subjected to the downstream analysis using Python software *scirpy* (version: 0.16.1). We assessed the productive TCR chain pairing status using function *scirpy.tl.chain_qc*, and defined clonotypes using functions *scirpy.pp.ir_dist* and *scirpy.tl.define_clonotypes*, depending on the CDR3 nucleotide sequence from TCR α- and β-chains as the primary identifier. The extent of clonotype expansion was quantified using function *scirpy.tl.clonal_expansion*. To examine the similarity of TCR repertoires across samples, we calculated and visualized the overlap using functions *scirpy.tl.repertoire_overlap* and *scirpy.pl.repertoire_overlap*, respectively. For each sample, TCR diversity was quantified using function

scirpy.tl.alpha_diversity with the diversity index D50.

References

[1] Q. Shi, Y. Chen, Y. Li, S. Qin, Y. Yang, Y. Gao, L. Zhu, D. Wang, Z. Zhang, Cross-tissue multicellular coordination and its rewiring in cancer, Nature 643 (2025) 529–538. https://doi.org/10.1038/s41586-025-09053-4.

[2] L. Zheng, S. Qin, W. Si, A. Wang, B. Xing, R. Gao, X. Ren, L. Wang, X. Wu, J. Zhang, N. Wu, N. Zhang, H. Zheng, H. Ouyang, K. Chen, Z. Bu, X. Hu, J. Ji, Z. Zhang, Pan-cancer single-cell landscape of tumor-infiltrating T cells, Science 374 (2021) abe6474. https://doi.org/10.1126/science.abe6474.

Figure S1

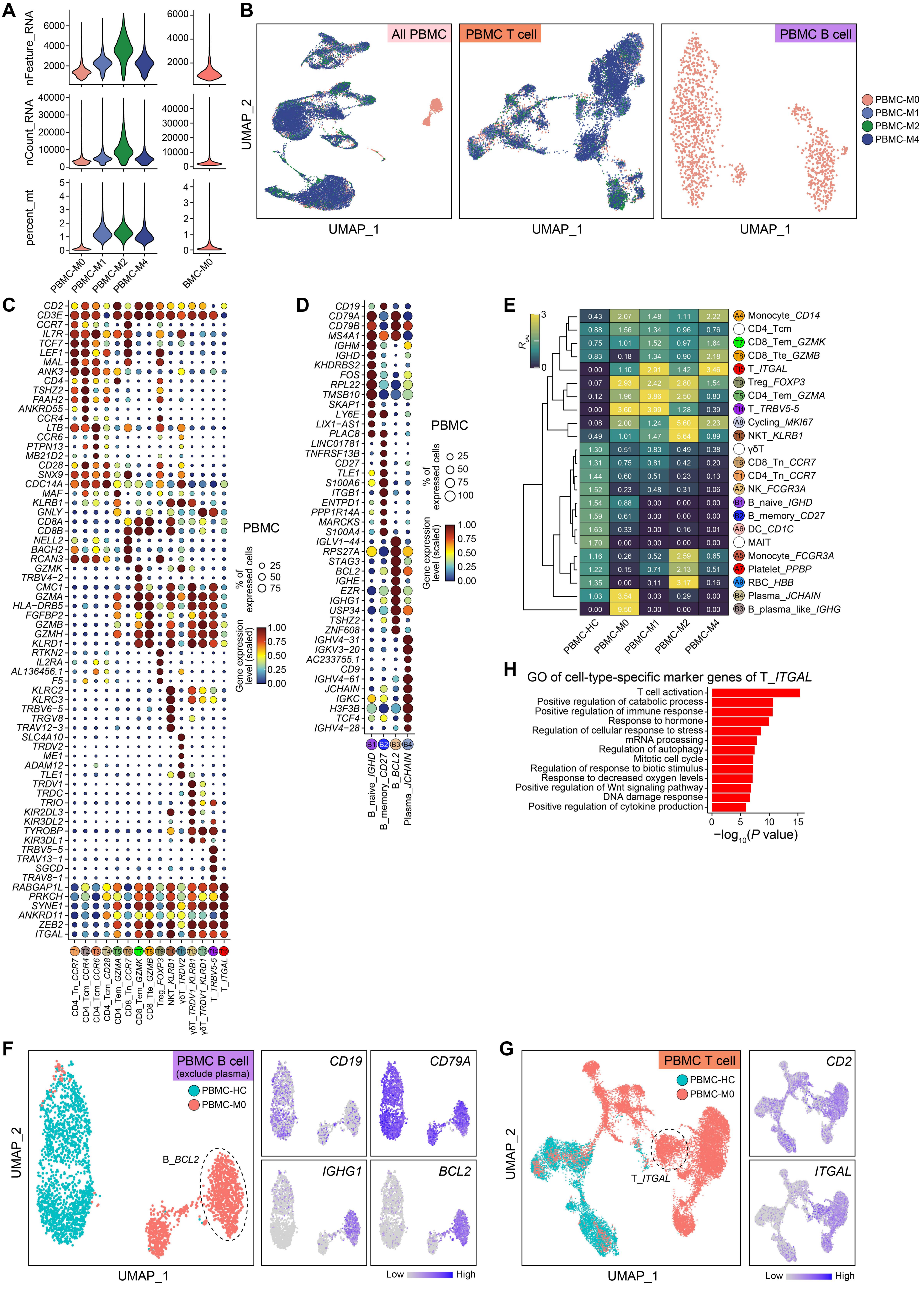

A
nFeature_RNA
nCount_RNA
percent_mt
PBMC-M0
PBMC-M1
PBMC-M2
PBMC-M4
BMC-M0
B
All PBMC
PBMC T cell
PBMC B cell
UMAP_2
UMAP_1
PBMC-M0
PBMC-M1
PBMC-M2
PBMC-M4
C
CD2
CD3E
CCR7
IL7R
TCF7
LEF1
MAL
ANK3
CD4
TSHZ2
FAAH2
ANKRD55
CCR4
LTB
CCR6
PTPN13
MB21D2
CD28
SNX9
CDC14A
MAF
KLRB1
GNLY
CD8A
CD8B
NELL2
BACH2
RCAN3
GZMK
TRBV4−2
CMC1
GZMA
HLA−DRB5
FGFBP2
GZMB
GZMH
KLRD1
RTKN2
IL2RA
AL136456.1
F5
KLRC2
KLRC3
TRBV6−5
TRGV8
TRAV12−3
SLC4A10
TRDV2
ME1
ADAM12
TLE1
TRDV1
TRDC
TRIO
KIR2DL3
KIR3DL2
TYROBP
KIR3DL1
TRBV5−5
TRAV13−1
SGCD
TRAV8−1
RABGAP1L
PRKCH
SYNE1
ANKRD11
ZEB2
ITGAL
PBMC
% of expressed cells
25
50
75
Gene expression level (scaled)
1.00
0.75
0.50
0.25
0.00
CD4_Tn_CCR7
CD4_Tcm_CCR4
CD4_Tcm_CCR6
CD4_Tcm_CD28
CD4_Tem_GZMA
CD8_Tn_CCR7
CD8_Tem_GZMK
CD8_Tte_GZMB
Treg_FOXP3
NKT_KLRB1
γδT_TRDV2
γδT_TRDV1_KLRB1
γδT_TRDV1_KLRD1
T_TRBV5-5
T_ITGAL
D
CD19
CD79A
CD79B
MS4A1
IGHM
IGHD
KHDRBS2
FOS
RPL22
TMSB10
SKAP1
LY6E
LIX1−AS1
PLAC8
LINC01781
TNFRSF13B
CD27
TLE1
ITGB1
ENTPD1
PPP1R14A
MARCKS
S100A6
S100A4
IGLV1−44
RPS27A
STAG3
BCL2
IGHE
EZR
IGHG1
USP34
TSHZ2
ZNF608
IGHV4−31
IGKV3−20
AC233755.1
CD9
IGHV4−61
JCHAIN
IGKC
H3F3B
TCF4
IGHV4−28
B_naive_IGHD
B_memory_CD27
B_BCL2
Plasma_JCHAIN
PBMC
% of expressed cells
25
50
75
100
Gene expression level (scaled)
1.00
0.75
0.50
0.25
0.00
E
R_o/e
3
0
0.43 2.07 1.48 1.11 2.22 Monocyte_CD14
0.88 1.56 1.34 0.96 0.76 CD4_Tcm
0.75 1.01 1.52 0.97 1.64 CD8_Tem_GZMK
0.83 0.18 1.34 0.90 2.18 CD8_Tte_GZMB
0.00 1.10 2.91 1.42 3.46 T_ITGAL
0.07 2.93 2.42 2.80 1.54 Treg_FOXP3
0.12 1.96 3.86 2.50 0.80 CD4_Tem_GZMA
0.00 3.60 3.99 1.28 0.39 T_TRBV5-5
0.08 2.00 1.24 5.60 2.23 Cycling_MKI67
0.49 1.01 1.47 5.64 0.89 NKT_KLRB1
1.30 0.51 0.83 0.49 0.38 γδT
1.31 0.75 0.81 0.42 0.20 CD8_Tn_CCR7
1.44 0.60 0.51 0.23 0.13 CD4_Tn_CCR7
1.52 0.23 0.48 0.31 0.06 NK_FCGR3A
1.54 0.88 0.00 0.00 0.00 B_naive_IGHD
1.59 0.61 0.00 0.00 0.00 B_memory_CD27
1.63 0.33 0.00 0.16 0.01 DC_CD1C
1.70 0.00 0.00 0.00 0.00 MAIT
1.16 0.26 0.52 2.59 0.65 Monocyte_FCGR3A
1.22 0.15 0.71 2.13 0.51 Platelet_PPBP
1.35 0.00 0.11 3.17 0.16 RBC_HBB
1.03 3.54 0.03 0.29 0.00 Plasma_JCHAIN
0.00 9.50 0.00 0.00 0.00 B_plasma_like_IGHG
PBMC-HC
PBMC-M0
PBMC-M1
PBMC-M2
PBMC-M4
H
GO of cell-type-specific marker genes of T_ITGAL
T cell activation
Positive regulation of catabolic process
Positive regulation of immune response
Response to hormone
Regulation of cellular response to stress
mRNA processing
Regulation of autophagy
Mitotic cell cycle
Regulation of response to biotic stimulus
Response to decreased oxygen levels
Positive regulation of Wnt signaling pathway
DNA damage response
Positive regulation of cytokine production
0 5 10 15
−log10(P value)
F
PBMC B cell (exclude plasma)
PBMC-HC
PBMC-M0
B_BCL2
UMAP_2
UMAP_1
CD19
CD79A
IGHG1
BCL2
Low
High
G
PBMC T cell
PBMC-HC
PBMC-M0
T_ITGAL
UMAP_2
UMAP_1
CD2
ITGAL
Low
High

Figure S2

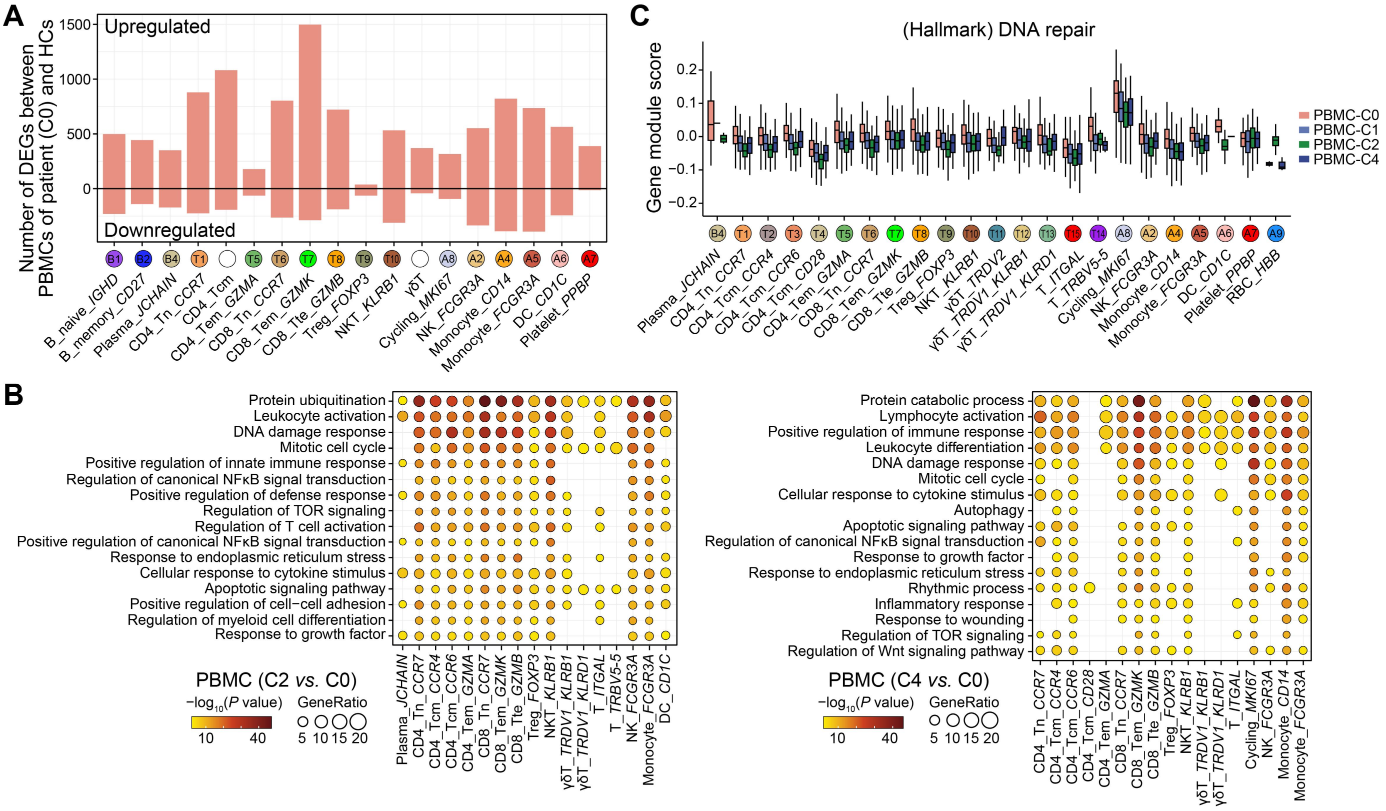

A
Upregulated
Downregulated
Number of DEGs between PBMCs of patient (C0) and HCs
B_naive_IGHD
B_memory_CD27
Plasma_JCHAIN
CD4_Tn_CCR7
CD4_Tcm
CD4_Tem_GZMA
CD8_Tn_CCR7
CD8_Tem_GZMK
CD8_Tte_GZMB
Treg_FOXP3
NKT_KLRB1
γδT
Cycling_MKI67
NK_FCGR3A
Monocyte_CD14
Monocyte_FCGR3A
DC_CD1C
Platelet_PPBP
C
(Hallmark) DNA repair
Gene module score
PBMC-C0
PBMC-C1
PBMC-C2
PBMC-C4
B
Protein ubiquitination
Leukocyte activation
DNA damage response
Mitotic cell cycle
Positive regulation of innate immune response
Regulation of canonical NFκB signal transduction
Positive regulation of defense response
Regulation of TOR signaling
Regulation of T cell activation
Positive regulation of canonical NFκB signal transduction
Response to endoplasmic reticulum stress
Cellular response to cytokine stimulus
Apoptotic signaling pathway
Positive regulation of cell−cell adhesion
Regulation of myeloid cell differentiation
Response to growth factor
PBMC (C2 vs. C0)
−log10(P value)
GeneRatio
Protein catabolic process
Lymphocyte activation
Positive regulation of immune response
Leukocyte differentiation
DNA damage response
Mitotic cell cycle
Cellular response to cytokine stimulus
Autophagy
Apoptotic signaling pathway
Regulation of canonical NFκB signal transduction
Response to growth factor
Response to endoplasmic reticulum stress
Rhythmic process
Inflammatory response
Response to wounding
Regulation of TOR signaling
Regulation of Wnt signaling pathway
PBMC (C4 vs. C0)

Figure S3

A

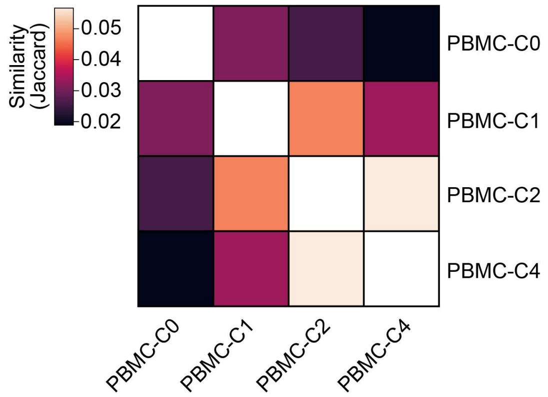

Similarity (Jaccard)
0.05
0.04
0.03
0.02
PBMC-C0
PBMC-C1
PBMC-C2
PBMC-C4
PBMC-C0
PBMC-C1
PBMC-C2
PBMC-C4

B

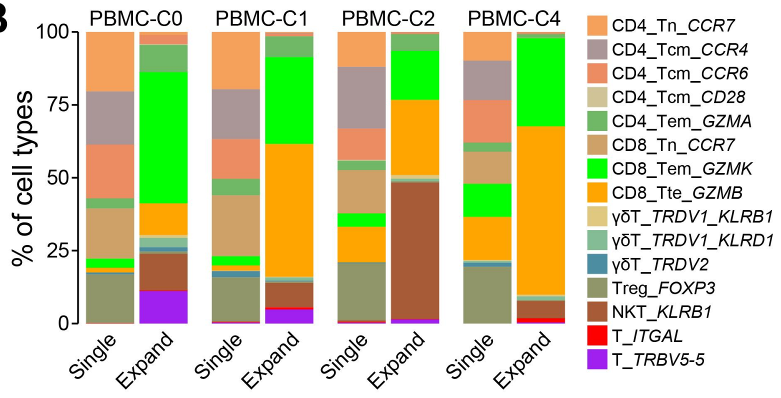

PBMC-C0
PBMC-C1
PBMC-C2
PBMC-C4
% of cell types
100
75
50
25
0
Single
Expand
Single
Expand
Single
Expand
Single
Expand
CD4_Tn_CCR7
CD4_Tcm_CCR4
CD4_Tcm_CCR6
CD4_Tcm_CD28
CD4_Tem_GZMA
CD8_Tn_CCR7
CD8_Tem_GZMK
CD8_Tte_GZMB
γδT_TRDV1_KLRB1
γδT_TRDV1_KLRD1
γδT_TRDV2
Treg_FOXP3
NKT_KLRB1
T_ITGAL
T_TRBV5-5
